# Extraversion Is a Mediator of Gelotophobia: A Study of Autism Spectrum Disorder and the Big Five

**DOI:** 10.3389/fpsyg.2018.00150

**Published:** 2018-02-20

**Authors:** Meng-Ning Tsai, Ching-Lin Wu, Lei-Pin Tseng, Chih-Pei An, Hsueh-Chih Chen

**Affiliations:** Department of Educational Psychology and Counseling, National Taiwan Normal University, Taipei, Taiwan

**Keywords:** autism spectrum disorder, laughter, gelotophobia, gelotophilia, katagelasticism, Big Five personality traits

## Abstract

Previous research has shown that individuals with autism are frequently mocked in their childhood and are consequently more anxious about being ridiculed. Research has also shown that autistic individuals have a higher level of gelotophobia (fear of being laughed at) compared to typically developed individuals. However, recent studies have also found that gelotophobia is strongly related to personality, which suggests that personality is a factor that helps to create a higher level of gelotophobia in autistic individuals. To investigate whether this is the case, we recruited 279 Taiwanese high school students, 123 with autism spectrum disorder (ASD) and 156 typically developed students as a control group. Self-reporting questionnaires were used to gather data on the Big Five personality traits and on the gelotophobia-related traits of gelotophobia, gelotophilia, and katagelasticism. The results were analyzed and the two groups were compared for differences in gelotophobia and personality. The ASD group was found to have a higher level of gelotophobia than the typically developed group, but lower levels of gelotophilia and katagelasticism. Additionally, the ASD group was found to have lower levels of extraversion and agreeableness than the typically developed group, but no significant difference was found between the two groups in terms of conscientiousness, openness, and emotional stability. We then investigated the possible correlations between gelotophobia-related traits and the Big Five, and consequently the mediation effect of the Big Five on gelotophobia. The results show, firstly, that extraversion rather than ASD is a direct factor in gelotophobia. Secondly, the level of gelotophilia was partly influenced by autism but also to a certain extent by the level of extraversion. Lastly, the results indicate that autism and the level of agreeableness are in conflict when predicting the level of katagelasticism.

## Introduction

When a person is teased or mocked by others, they usually experience negative feelings such as anger, sadness, shame, disgust, or fear (Platt, 2008; Platt and Ruch, 2009). Generally, however, most people can cope with such situations and modify their responses accordingly (Chen et al., 2011). Nevertheless, there remain some people who cannot tell the difference between playful teasing and malicious ridicule. Such individuals perceive all jokes to be hostile and cannot respond lightheartedly or cheerfully to jokes or laughter when interacting socially (Titze, 2009). People who are paranoid about laughter have *gelotophobia*—a fear of being laughed at (Ruch and Proyer, 2008a). They are sensitive to laughter in all social situations, dread being laughed at, and regard others' smiles only as scornful (Hofmann et al., 2015). Their extreme fear results in maladjusted behaviors (Ruch and Proyer, 2009a), certain cases of which are often the subject of clinical research (Titze, 1996, 2009; Ruch et al., 2009). The term “gelotophobia” (from *gelos*, Greek for laughter) was proposed by Titze (1995, 1996, 1997). Based on his clinical observations, some people seem to be excessively concerned about being laughed at by others. They cannot distinguish the difference between playful teasing and ridicule, and perceive all types of laughter as hostile (Titze, 1995, 1996, 1997). Titze (2009) claimed that gelotophobes might be accustomed to seeing poker-faced or apathetic expressions in childhood, which leads them to respond fearfully to any jokes, even to good-humored, playful banter. In such circumstances, gelotophobes allay their state of uneasiness non-verbally; out of anxiety, their faces become stiff (Titze, 2009).

In a series of empirical studies, Ruch and his colleagues (Ruch and Proyer, 2008a,b, 2009a,b; Ruch et al., 2010, 2014) found that gelotophobia was not only seen among otherwise healthy people but also more generally in different cultures, and they developed two other concepts that were related to laughter, but were contrary to the fear of being laughed at Ruch and Proyer (2009a) described two emotional aspects of laughter (fear and joy) and their relationship to laughter's object (the self and others). Thus they found three related traits: gelotophobia, where the individual fears being laughed at; gelotophilia, where the individual enjoys being laughed at; and katagelasticism, where the individual enjoys laughing at others. In contrast to gelotophobes, “gelotophiles” feel positive and happy when they are being laughed at. They are not fearful or afraid of being ridiculed; in fact, they enjoy it when others laugh at them. Moreover, they actively seek situations in which others may laugh at them; for example, by sharing embarrassing things that have happened to them, or speaking openly about misfortunes and mishaps, thus provoking laughter in their audience. It is noticeable that gelotophobes and gelotophiles are not two extremes of the same type; rather, they are two distinct types. Their responses to being ridiculed are opposite. Gelotophiles are not only unafraid of being laughed at, but also gain pleasure from it. Gelotophobes and gelotophiles exhibit entirely different characteristics (Ruch and Proyer, 2009a). Besides self-directed laughter, Ruch and Proyer (2009a) found that some people seek situations in which they can laugh at others; such individuals are termed “katagelasticists.” Katagelasticists initially look for the chance to mock others and then revel in seeing others fall victim to embarrassing or unfortunate events; furthermore, they continue to search for any opportunity to ridicule these same people, and make fun of them by insulting them directly or by using offensive words to describe them. In addition, katagelasticists never make fun of themselves to please others, and will defend themselves if others laugh at them.

As for the causes of gelotophobia, Ruch (2004, 2009) proposed the “model of the putative causes and consequences of gelotophobia,” which was later revised (Titze, 2009; Ruch et al., 2014). He claimed that children who did not feel loved or appreciated within the parent–child relationship do not develop a sense of belonging, and then withdraw socially to avoid being ridiculed. The experience of being mocked is possibly the origin of their fear of being laughed at, and this fear extends into adulthood, as expressed by the styles identified in the PhoPhiKat questionnaire (Chen et al., 2011). According to the study conducted by Platt and Ruch (2009), a positive correlation exists between the experience of being bullied and gelotophobia. Moreover, a study by Samson et al. (2011) also confirmed an association between gelotophobia and a past experience of ridicule. All of these studies revealed that the fear of being laughed is more marked when the past experience of being derided or bullied was serious or frequent, and that it is hard to “shake off” such a fear once it has taken root (Liu et al., 2014). The revision of this model included the external conditions and internal factors about the fear of being laughed at, where external conditions referred to the peer group norm, societal structure, cultural factors and so forth, and internal factors referred to genetics, personality, emotional dispositions and so on (Ruch et al., 2014). The revision of the model indicates that not only early experience, but also personality, social skills, and external conditions, are all potential factors in the development of gelotophobia. The revised model is more effective at explaining the cause–effect relationship of gelotophobia and how the findings can be used to help individuals.

Research into the reasons for, and prevention of, gelotophobia indicates that individuals with gelotophobia usually have problems with emotional adjustment and social skills (Papousek et al., 2009, 2014). It is also seen among some patients with psychological defects (Weiss et al., 2012); in particular, some researchers are interested in the connection between autism spectrum disorder (ASD) and gelotophobia (Samson et al., 2011; Wu et al., 2015).

ASD is a neurodevelopmental disorder that is usually characterized by weak social and communication skills, along with the tendency to show fixed repeated behaviors and activities (APA, 2013). For those with autism, such weaknesses affect their social functioning and the ability to empathize, which makes it extremely difficult for them to recognize, or identify with, the mindset of other people, including their beliefs, thoughts, and emotions (Baron-Cohen et al., 1985; Baron-Cohen, 1989, 2001). In addition, such individuals also find it difficult to interpret the non-verbal cues of other people, such as body language (Asperger, 1944; Attwood, 2000). The lack of these interpersonal skills means that peers perceive those with autism as odd, unsociable, stubborn, and self-centered. Past research has claimed that when those with autism fail to grasp the latent agreement or implicit rules within group interactions, they become frustrated and experience increasing psychological pressure, which then results in emotional disturbance (Myles and Simpson, 2003; Attwood, 2007). Some such individuals even tend to use aggressive humor (Wu et al., 2014), which can lead to those with autism being singled out or teased.

Children with autism are frequently mocked or teased for their clumsy or odd behaviors (Carter, 2009). They do not know how to make friends and consequently become isolated from their peers. Samson et al. (2011) indicated that people with autism are very fearful of being ridiculed owing to an early experience of being bullied and being laughed at. The study by Samson et al. (2011) showed that the proportion of people with gelotophobia was higher among the autism group (45%) than among the typically developed group (6%). The level of gelotophobia was positively correlated with the frequency of being ridiculed and the severity of the ridicule. In addition, people with autism did not like self-ridiculing (i.e., they had a low level of gelotophilia), but liked to laugh at others, as did those in the typically developed group. Similar results have been found in a study of Taiwanese people (Wu et al., 2015): those with autism exhibited a higher level of gelotophobia but a lower level of gelotophilia. Moreover, in terms of the level of katagelasticism, no significant difference was found between the Taiwanese individuals with autism and the typically developed group (Wu et al., 2015).

However, while those with autism have a higher level of gelotophobia than the level seen in a typically developed group, individual difference is significant: not all those with autism have gelotophobia (Wu et al., 2015). To understand why some people with ASD have gelotophobia while others with ASD do not, researchers have investigated the relationship between personality and gelotophobia.

Personality is stable and built into the early stages of life, and is usually considered a higher hierarchical trait (Furnham, 2008). Many researchers concur that personality could explain why only some children experience emotional and behavioral problems (Eaves et al., 1994; Wing, 1997; Hepburn, 2003; Leyfer et al., 2006). An individual usually shows a disposition in early childhood (Rothbart et al., 2006), and research has revealed that personality is generally relevant to maladapted behaviors, both in individuals with autism and in typically developed individuals (Mervielde et al., 2005, 2006). Previous research has revealed that personality and the fear of being laughed at are related traits, but discussions about the causes of gelotophobia in those with autism have almost exclusively focused on the influence of childhood experience; for example, early experiences of being mocked (Samson et al., 2011; Proyer and Neukom, 2013) and parental attachment (Wu et al., 2015).

However, the literature has also highlighted a strong association between gelotophobia and personality: since personality determines how individuals cope with social situations and subsequent events, and since gelotophobia is related to many sub-factors, such as fear and anxiety, personality is also a source of gelotophobia (Ruch and Proyer, 2009b). Ruch et al. (2013) found that gelotophobia could be predicted by the personal traits of the individual, and was also correlated to the “Big Five” personality traits. In particular, gelotophobia was positively correlated with neuroticism, but negatively correlated with extraversion and an openness to experience. Gelotophilia was found to be positively correlated with extraversion and openness, but negatively correlated with conscientiousness and neuroticism. Finally, katagelasticism was found to be negatively correlated with levels of agreeableness and conscientiousness. Other research has revealed similar findings (Chen et al., 2011; Proyer et al., 2012a,b,c). Studies that have investigated the personalities of those with ASD and those in a typically developed group also found that the level of neuroticism was higher in those with autism than in those in the typically developed group, while the levels of extraversion, agreeableness, openness, and conscientiousness were lower; these findings were consistent for both children and adults, male or female (Schriber et al., 2014). In their revised model of gelotophobia, Ruch et al. (2014) claim that personality is an antecedent risk factor for gelotophobia. We assume therefore that personality may be the key trait of gelotophobia in those with ASD.

To determine the potential causes of gelotophobia, the present study aims to understand the connection between the tendency toward gelotophobia in individuals with autism, and personality. We investigated the difference in the degree of gelotophobia between those with autism and a typically developed group, and how personality mediates the extent of gelotophobia. As teenagers with autism are more likely to be interacting with peers at school, and thus to encounter situations in which they are laughed at, or they laugh at others (Van Roekel et al., 2010), the present study focused on high school students.

**Figure 1:**
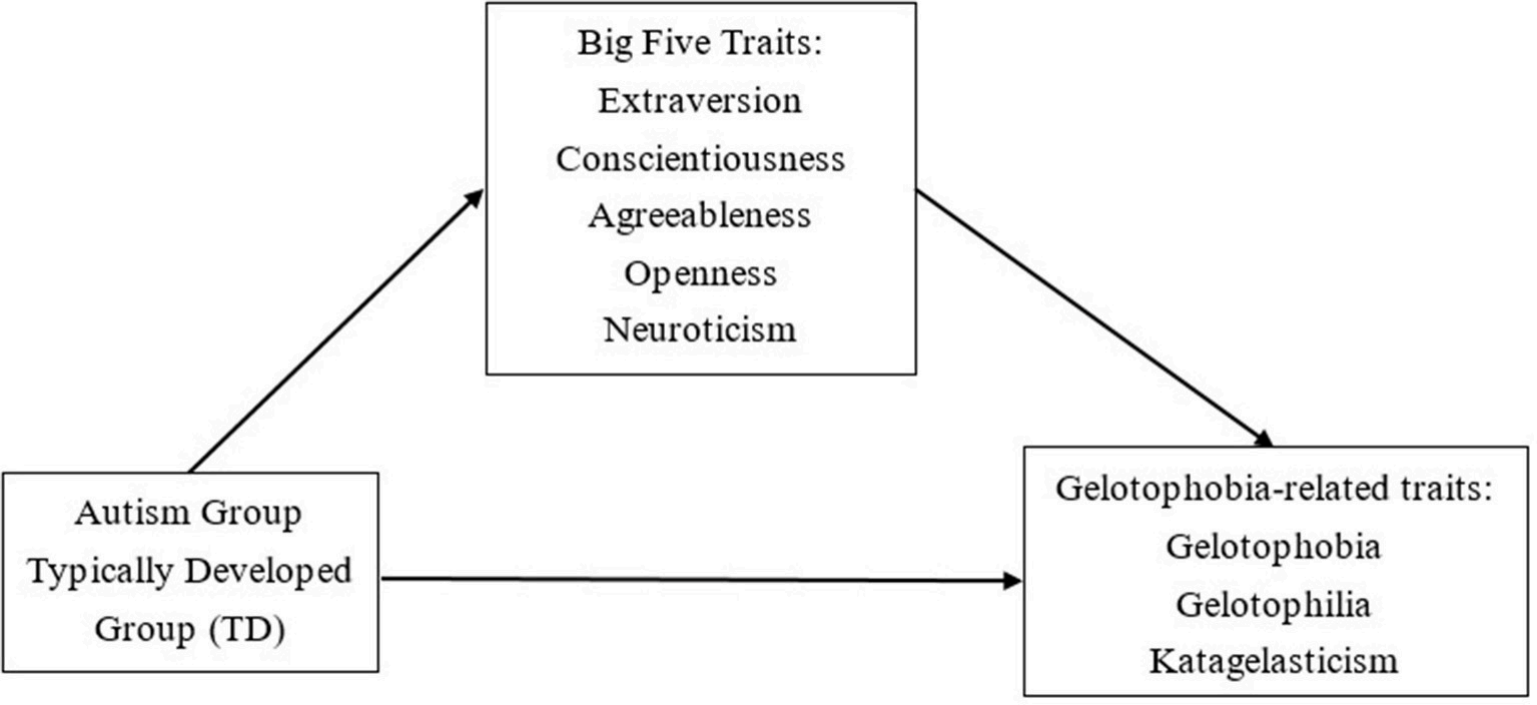
Research structure.

We investigated the differences between the Big Five personality traits and gelotophobia among teenagers with autism and without autism, and then investigated the relationship between the mediating effects of the Big Five personality traits and gelotophobia in the autism group and the typically developed group. The research structure is shown in Figure 1. We duplicated past research by establishing two groups as independent variables (teenagers with autism vs. the typically developed teenagers as the control group) and traits for fear of being laughed at (gelotophobia, gelotophilia, and katagelasticism) as dependent variables, with the Big Five personality traits as mediators between the two sets of variables.

We hypothesized that teenagers with autism would have a greater tendency toward gelotophobia and would have a greater dislike of being laughed at than the members of the typically developed group. The study conducted by Ruch and Proyer (2009b) found that gelotophobia was positively related to extraversion but negatively related to neuroticism as defined in Eysenck's psychoticism, extraversion, and neuroticism (PEN) model of personality (Eysenck et al., 1985); therefore, we also hypothesized that extraversion and neuroticism were associated with a tendency toward gelotophobia for individuals with autism.

## Materials and methods

### Participants

The present study recruited students with autism from high schools in Taiwan. These students had been diagnosed by doctors or by the municipal special education identification and counseling committees composed of special education professionals, who confirmed the presence of autism or Asperger syndrome as defined by the following *Diagnostic and Statistical Manual of Mental Disorders, Fifth Edition* (DSM-V) criteria: (1) having notable impaired verbal and non-verbal communication; (2) having notable impaired social interaction; (3) having restricted and repetitive behavior; and (4) having intelligence quotients (IQs) of 70 or above according to the Wechsler Intelligence Scale for Children. For the typically developed group, we recruited high school students without autism from the same area as the high school students with autism. Excluding invalid questionnaires (i.e., those with a ratio > 1:8 of incomplete material or inconsistent answers), the sample group comprised 123 high school students with autism while the typically developed group comprised 156 high school students without autism; the total sample size was 279 participants. In the autism group, 87% of the participants were male, which matches the gender ratio (8:1 male to female) of individuals with autism in Taiwan aged 14–18 years (*M* = 15.67, *SD* = 1.33). In the typically developed group, 87% of the participants were again male, but aged 15–18 years (*M* = 15.84, *SD* = 0.65). There were no differences in gender or age between the two groups [*X*^2^ = 0.002, *p* = 0.963; *t*_(277)_ = 1.361, *p* = 0.175]. The study was approved by the Institution Review Board of Taipei Medical University. All participants were informed of the study procedure and provided informed consent before commencement of the study.

### Materials

The research tools were the “Big Five mini-markers” and the PhoPhiKat-TC scale. These are described below.

#### Big five mini-markers

We used the Big Five mini-markers test employed in the study by Chen et al. (2011). This study was translated into Chinese by Saucier (1994). It includes five constructs: *extraversion, conscientiousness, agreeableness, openness*, and *emotional stability*. Saucier used *emotional stability* to replace *neuroticism* to ensure consistency between the five constructs. Every construct is composed of eight adjectives, totaling 40 items. The participants rate each item using a seven-point scale; a higher rating was recorded when the participant believed the item to be consistent with their own characteristics. The results of factor analysis by Saucier (1994) showed that the loading for all factors was >0.40, and the Cronbach's alpha for the five factors ranged from 0.76 to 0.85. Both reliability and validity were found to be satisfactory.

#### PhoPhiKat-TC scale

We used the PhoPhiKat-TC scale described by Chen et al. (2011). This scale encompasses three concepts: gelotophobia, gelotophilia, and katagelasticism. Every concept is measured by 15 items, and the entire scale comprises 45 items. The participants rate each item on a four-point scale; the more the participants agree with the item, the higher they rate it. With regard to the construct validity, the indices of model fit were found to be higher than 0.90 according to the results of confirmatory factor analysis. The construct validities of the three factors were found to be satisfactory. The Cronbach's alpha of each sub-scale was 0.85, and the test-retest reliability ranged from 0.87 to 0.92. The reliability of the scale was also found to be satisfactory.

### Procedure

At the initial stage of the research, we investigated the distribution of Taiwanese high school students with autism. We invited the relevant high schools to survey their students regarding their willingness to participate in the research. We then recruited the participants and carried out the research in the high schools where the students with autism were studying; all participants received detailed information about the content of the research and provided their written consent prior to participation. Research was implemented by means of the test and the questionnaire. Researchers first introduced the purpose of the research and offered guidance on how to fill in the questionnaire. When all the participants fully understood the procedure, they began to complete the Big Five mini-markers test and the PhoPhiKat-TC questionnaire in sequential order. The total time for completing the questionnaire was 15 min. All the participants received a set of stationery items upon completion.

## Results

### Comparison of gelotophobia in students with ASD and those in the typically developed group

To classify the level of gelotophobia, Ruch and Proyer (2008b) designated the cut-off points of the PhoPhiKat as follows: no gelotophobia 1.0–2.5; slight gelotophobia 2.5–3.0; marked gelotophobia 3.0–3.5; and extreme gelotophobia 3.5–4.0. The result of a Chi-square test revealed a significant difference in gelotophobia between the two groups (*X*^2^ = 8.597, *p* = 0.035): 73.7% of the typically developed group had no gelotophobia, compared with 57.7% of the ASD group; and 35.8% of the ASD group had slight gelotophobia, compared with 20.5% in the typically developed group. No difference was found between the ASD group and the typically developed group with regard to extreme gelotophobia (Table 1).

**Table 1:** Comparison of gelotophobia in ASD group and typically developed group.

			**Group**		**Total**
			**TD**	**ASD**	
Level of gelotophobia	No	Count	115^a^	71^b^	186
		% within group	73.7	57.7	66.7
	Slight	Count	32^a^	44^b^	76
		% within group	20.5	35.8	27.2
	Marked	Count	7^a^	6^a^	13
		% within group	4.5	4.9	4.7
	Extreme	Count	2^a^	2^a^	4
		% within group	1.3	1.6	1.4
Sub-total		Count	156	123	279
		% within group	100.0	100.0	100.0

a,b *Significant difference between ASD group and typically developed group, p < 0.05*.

### Differences in personality traits and gelotophobia-related traits

The multivariate analysis of variance (MANOVA) results are reported in Table 2. In terms of the traits related to gelotophobia, the ASD group had a higher level of gelotophobia than the typically developed group [*F*_(1, 277)_ = 3.938, *p* = 0.048, η^2^ = 0.14], but lower levels of gelotophilia [*F*_(1, 277)_ = 47.752, *p* < 0.001, η^2^ = 0.147] and katagelasticism [*F*_(1, 277)_ = 5.208, *p* = 0.023, η^2^ = 0.18] than the typically developed group.

**Table 2:** Mean and SD of Big Five and gelotophobia-related traits between groups.

	**ASD group *M(SD) N* = 123**	**TD group *M(SD) N* = 156**	***F***	***P***	**η^2^**
**PHOPHIKAT-TC SCALE**					
Gelotophobia	2.42 (0.48)	2.30 (0.47)	3.938^*^	0.048	0.014
Gelotophilia	1.98 (0.47)	2.38 (0.50)	47.752^**^	<0.001	0.147
Katagelasticism	2.00 (0.50)	2.13 (0.47)	5.208^*^	0.023	0.018
**BIG FIVE PERSONALITY**					
Extraversion	3.85 (0.99)	4.34 (1.11)	14.604^**^	<0.001	0.050
Conscientiousness	3.76 (0.83)	3.94 (0.83)	3.578	0.060	0.013
Agreeableness	4.56 (0.91)	4.97 (0.89)	14.344^**^	<0.001	0.049
Openness	4.36 (0.94)	4.55 (0.95)	2.788	0.096	0.010
Emotional stability	3.91 (0.97)	4.10 (0.95)	2.764	0.098	0.010

* *p < 0.05*,

** *p < 0.01*.

Regarding personality, the ASD group was found to have a lower level of extraversion [*F*_(1, 277)_ = 14.604, *p* < 0.001, η^2^ = 0.050] and agreeableness [*F*_(1, 277)_ = 14.344, *p* < 0.001, η^2^ = 0.049] than the typically developed group; but no significant difference was found between the two groups in terms of conscientiousness, openness, and emotional stability.

### Canonical correlation of gelotophobia-related traits and personality

To investigate the correlation between gelotophobia-related traits and the Big Five, as well as to diminish the interference between traits, we used a canonical correlation to determine the difference between the Big Five and gelotophobia-related traits both within groups and between groups. According to our hypotheses, personality is the antecedent variable of gelotophobia; therefore, Big Five personality traits are predictive variables, and gelotophobia-related traits are criterion variables.

The results are shown in Table 3. Within the typically developed group, for Big Five and gelotophobia-related traits, three sets of generalized *F* coefficients of roots were found to be significant. The coefficients of canonical correlation for the three sets were 0.666 (*p* < 0.001), 0.543 (*p* < 0.001), and 0.380 (*p* = 0.003). In order, the first coefficient indicates that extraversion (−0.863) had an influence on the level of gelotophobia (0.971) (the lower the level of extraversion, the higher the gelotophobia); the second coefficient indicates that agreeableness (0.789) had an influence on the level of katagelasticism (−0.983) (the higher the level of agreeableness, the lower the katagelasticism); and the third coefficient indicates that emotional stability (0.563) had an influence on the level of gelotophilia (−0.889) (the higher the level of emotional stability, the lower the gelotophilia).

**Table 3:** Correlation of Big Five and gelotophobia-related traits.

**Group**	**TD**			**ASD**		
**Root**	**1**	**2**	**3**	**1**	**2**	**3**
Eigenvalue	0.796	0.419	0.098	0.645	0.312	0.051
Variance	60.593	31.891	7.517	63.954	30.941	5.104
Canonical	0.666	0.543	0.380	0.626	0.488	0.221
Wilks	0.357	0.642	0.910	0.441	0.725	0.951
*F*	12.320	9.258	4.936	7.326	5.063	2.008
*p*	<0.001	<0.001	0.003	<0.001	<0.001	0.117
**PREDICTIVE VARIABLES**						
Extraversion	**−0.863**	−0.206	−0.208	0.103	**−0.901**	
Conscientiousness	−0.206	0.633	0.148	−0.475	−0.282	
Agreeableness	−0.492	**0.789**	−0.291	**−0.966**	−0.062	
Openness	−0.530	−0.006	−0.487	−0.051	−0.456	
Emotional stability	−0.612	0.480	**0.563**	−0.695	−0.277	
**CRITERION VARIABLES**						
Gelotophobia	**0.971**	−0.018	−0.240	0.176	**0.775**	
Gelotophilia	−0.320	−0.327	**−0.889**	0.042	−0.575	
Katagelasticism	0.142	**−0.983**	−0.117	**0.891**	−0.028	

^*^p < 0.05, ^**^p < 0.01. The bold values are highest correlation in the root.

Within the ASD group, for Big Five and gelotophobia-related traits, two sets of generalized F coefficients of roots were found to be significant. The coefficients of canonical correlation for the two sets were 0.626 (*p* < 0.001) and 0.488 (*p* < 0.001). In order, the first coefficient indicates that agreeableness (−0.966) had an influence on the level of katagelasticism (0.891) (the lower the level of agreeableness, the higher the katagelasticism); and the second coefficient indicates that extraversion (−0.901) had an influence on the level of gelotophobia (0.775) (the lower the extraversion, the higher the gelotophobia).

In terms of the results of the canonical correlation for Big Five and gelotophobia-related traits, similar findings were recorded for both groups; i.e., that extraversion is the best predictor of gelotophobia, and agreeableness is the best predictor of katagelasticism. The main difference between the groups was that emotional stability was proven to be the best predictor of gelotophobia for the typically developed group, but not for the ASD group.

### Mediation analysis of the big five personality markers

To investigate the mediation effect of the Big Five personality markers on gelotophobia for people with autism and the typically developed group, we used gender and age as the control variables and the group (the autism group vs. the typically developed group) as the independent variable. For the dummy coding of the group, those with autism were assigned a value of 1 and those in the typically developed group were assigned a value of 0. We then used the Big Five personality markers as mediators and performed a mediation analysis using gelotophobia, gelotophilia, and katagelasticism as dependent variables. For the mediation analysis, we used a bootstrapping approach (Preacher and Hayes, 2008), which simulates the data of a large sample by re-sampling from current data. Analyzing a large sample of data means that we can obtain a more precise prediction. In the present study, our simulation sample comprised 5,000 people. First, we made a prediction for the groups in terms of personality, and then we investigated the predictive power of personality by using gelotophobia, gelotophilia, and katagelasticism as dependent variables. Finally, we analyzed the predictions for the groups in terms of gelotophobia, gelotophilia, and katagelasticism, and the change in the predictions after using personality as a mediator.

#### Predictions for groups in terms of personality

Because the predictive power of groups in terms of personality is not affected by using gelotophobia, gelotophilia, and katagelasticism as dependent variables, we began the analysis with a path analysis. The findings revealed that groups are powerful in terms of predicting extraversion (β = −0.499, *p* < 0.01) and agreeableness (β = −0.394, *p* < 0.01). The β-values were negative for both groups and indicated that individuals with autism have lower levels of extraversion and agreeableness.

#### Using gelotophobia as a dependent variable

Only extraversion (β = −0.171, *p* < 0.001) and emotional stability (β = −0.175, *p* < 0.001) were found to be statistically significant with regard to predicting gelotophobia by personality. In other words, those with lower levels of extraversion and emotional stability had higher levels of gelotophobia.

**Figure 2:**
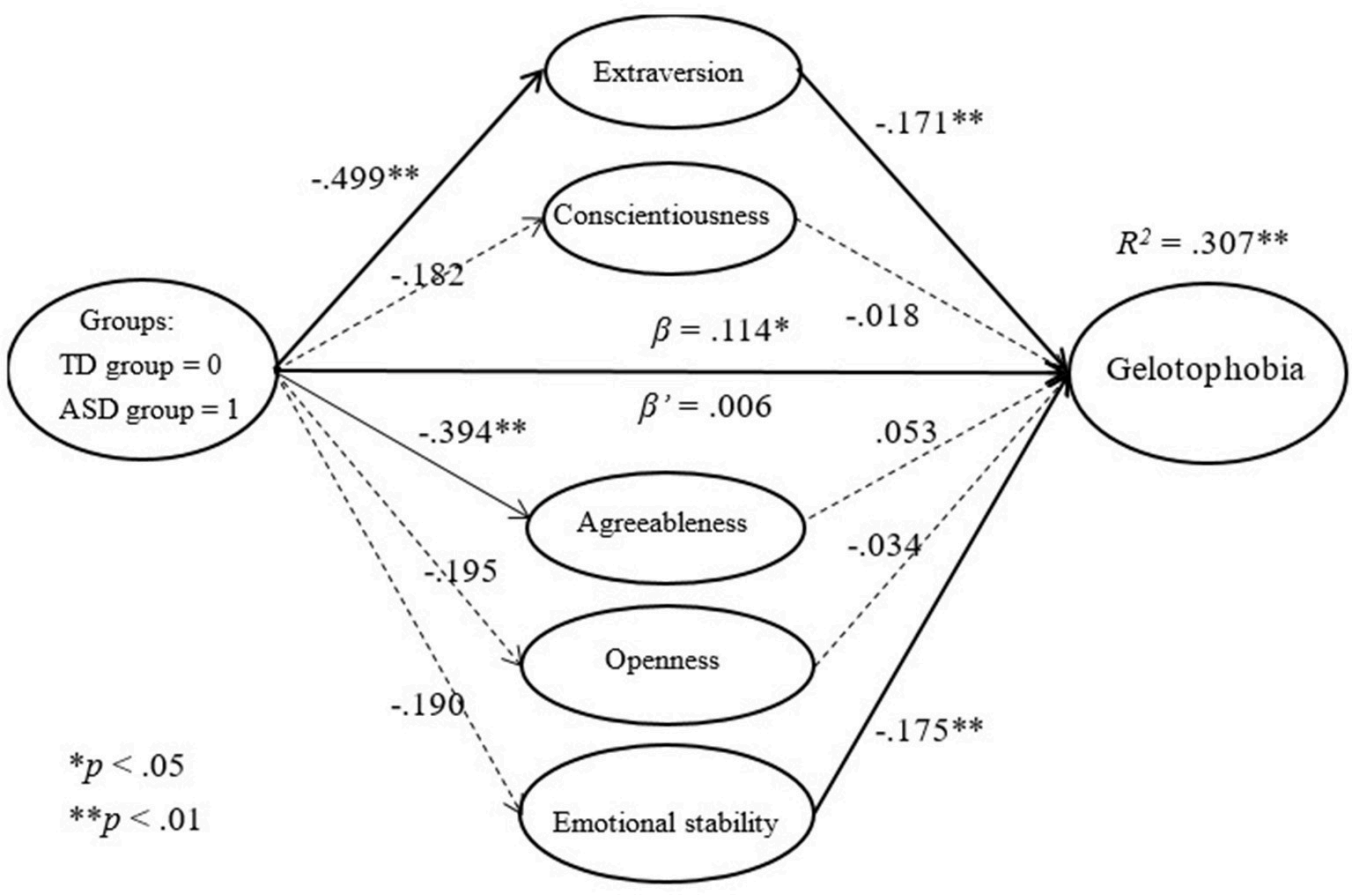
The mediation effect of personality on gelotophobia.

Concerning the predictions for groups in terms of gelotophobia and the change in the prediction after using personality as a mediator, the findings revealed that groups significantly predict the level of gelotophobia (β = 0.114, *p* = 0.047). However, after using personality as a mediator, the power of the groups disappears (β = 0.006, *p* = 0.910), and the power of prediction is completely overtaken by personality. Upon further examination, the mediation effect of personality was found to derive mainly from extraversion (95% CI [0.041, 0.145]) and shows that gelotophobia is significantly influenced by extraversion; i.e., individuals with lower levels of extraversion are more fearful of being laughed at (Figure 2). To conclude, extraversion, rather than the groups (with or without autism), influences the level of gelotophobia.

#### Using gelotophilia as a dependent variable

Our findings showed that extraversion (β = 0.105, *p* < 0.001) and openness (β = 0.081, *p* = 0.016) were found to be significant in predicting gelotophilia. Individuals with higher levels of extraversion or openness had higher levels of gelotophilia.

**Figure 3:**
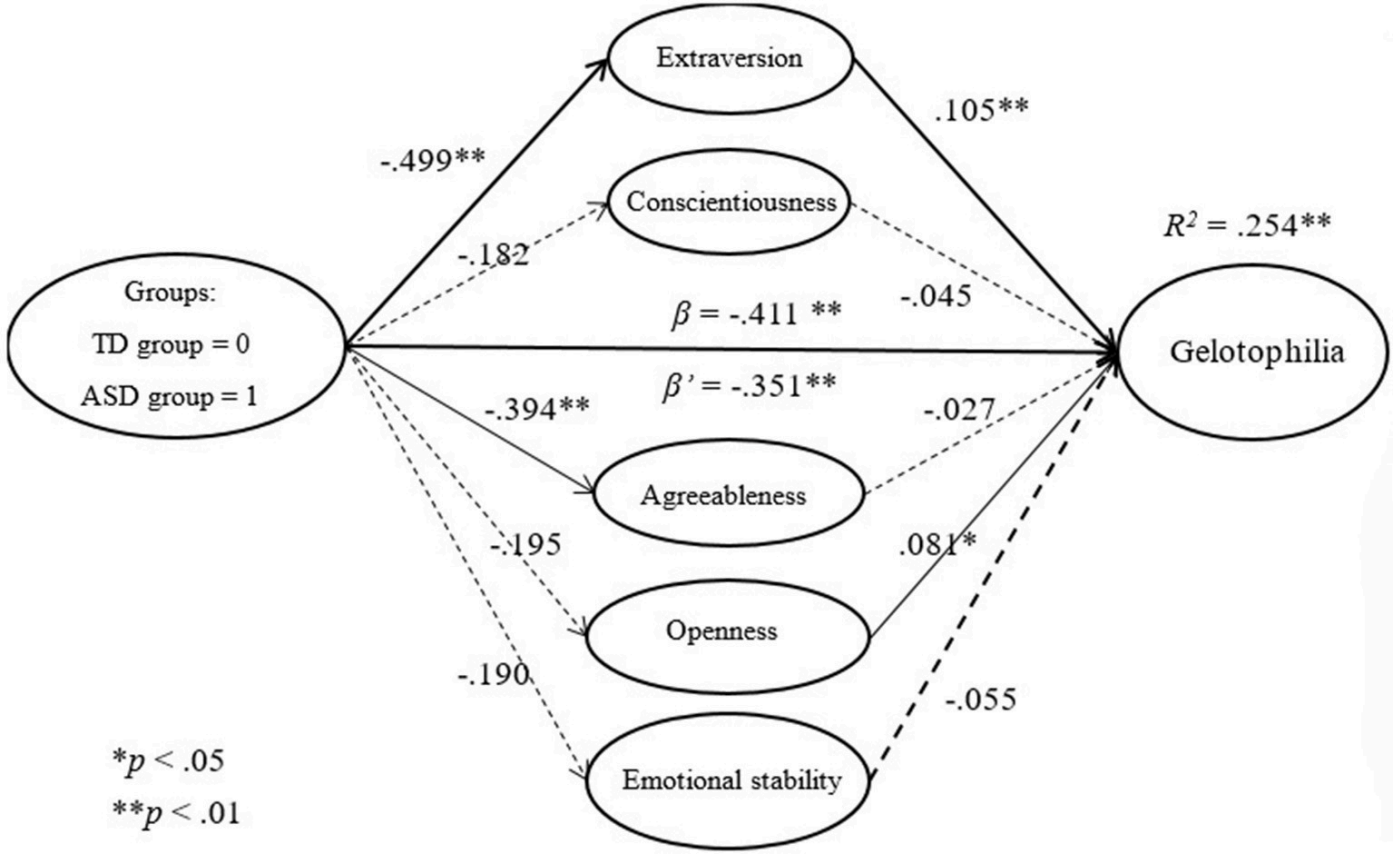
The mediation effect of personality on gelotophilia.

We then investigated the predictions for groups and the change after using personality as a mediator. Our results showed that the group significantly predicted the level of gelotophilia (β = −0.411, *p* < 0.001). The group effect remains but decreases after using personality as a mediator (β = −0.351, *p* < 0.01). The Sobel test clearly showed statistical significance (*p* < 0.05), and the mediation effect of personality was only partial. The mediation effect of personality was found to derive from extraversion (95% CI [−0.106, −0.015]). In summary, those with autism had lower levels of gelotophilia, as did those with lower levels of extraversion (as shown in Figure 3).

#### Using katagelasticism as a dependent variable

Extraversion (β = 0.063, *p* = 0.015), conscientiousness (β = −0.085, *p* = 0.012), agreeableness (β = −0.219, *p* < 0.001), and emotional stability (β = −0.065, *p* = 0.044) were found to be statistically significant in predicting the level of katagelasticism. Individuals with higher levels of extraversion had higher levels of katagelasticism. In contrast, individuals with higher levels of conscientiousness, agreeableness, and emotional stability had lower levels of katagelasticism.

**Figure 4:**
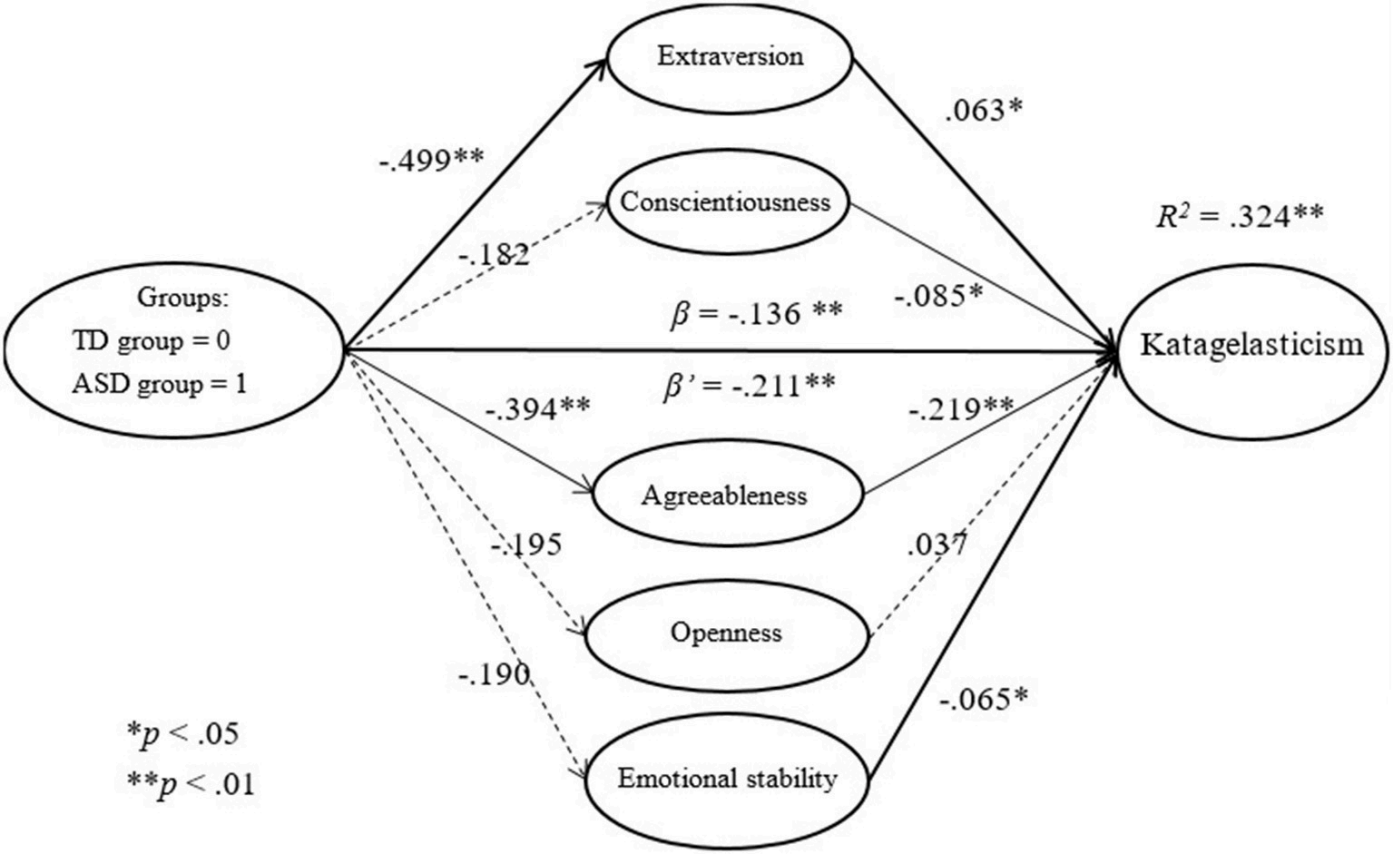
The mediation effect of personality on katagelasticism.

Concerning the predictions for groups in terms of katagelasticism and the change after using personality as a mediator, our findings show that the group is powerful in predicting the level of katagelasticism (β = −0.136, *p* = 0.021); moreover, its power slightly increases after using personality as a mediator (β = −0.211, *p* < 0.001). Personality is not a mediator, but rather a suppressor. Upon further analysis, the suppression effect of personality was found to derive from agreeableness (95% CI [0.041, 0.151]). Individuals with lower levels of agreeableness had higher levels of katagelasticism. Those with autism were found to have lower levels of agreeableness, but also lower levels of katagelasticism, than those in the typically developed group. Our results indicate that autism and the level of agreeableness are in conflict when predicting the level of katagelasticism. After using personality as a mediator, the effect of the group is more powerful in predicting the level of katagelasticism (as shown in Figure 4).

## Discussion

### Conclusion and implications

Past research has indicated that early experience affects whether gelotophobia develops in those with autism. Such individuals were thought to be lacking in communication and empathy skills and were often ridiculed by peers, which ultimately resulted in a fear of being laughed at. Personality was seldom considered as a contributory factor in terms of the fear of being laughed at in those with autism. Moreover, in the earlier studies of the personalities of those with autism, and the associations of personality with gelotophobia, the sample sizes were usually fewer than 50 participants, such as the research undertaken by Samson et al. (2011) and Schriber et al. (2014).

The present study recruited participants from high schools in Taiwan, selecting a typically developed group of teenagers and a group of teenagers with autism. We used a self-reporting questionnaire as a research tool, and investigated the difference in the level of gelotophobia between those teenagers with autism and those without. We then considered personality as a mediator to verify the relationship of autism to gelotophobia.

Our findings show that those individuals with ASD had higher levels of gelotophobia than those in the typically developed group. Further, we found that the most significant difference between the two groups was that the ASD group had a higher percentage of slight gelotophobia, and a lower percentage of no gelotophobia, than those in the typically developed group. In general, those with ASD had a higher level of gelotophobia than those in the typically developed group, particularly at the “slight” level, though no difference was found between the “marked” level and the “extreme” level between the two groups. The ASD group was found to have lower levels of katagelasticism and gelotophilia; i.e., those with ASD dislike being laughed at, but also are not interested in laughing at others. This finding is consistent with the findings of previous research (Wu et al., 2015).

Regarding personality, our results indicate that those with ASD had lower levels of extraversion and agreeableness than those in the typically developed group, possibly due to the fact that Chinese culture strongly emphasizes interpersonal harmony (Markus and Kitayama, 2010). Individuals with ASD tend to have poor social skills; consequently, those in the ASD group lagged behind those in the typically developed group in certain situations. Thus the difference between the ASD group and the typically developed group can perhaps be explained by cultural factors, and suggests that cultural factors should be taken into account in future studies. The other difference between the ASD group and the typically developed group was the canonical correlation results. We found that, for both groups, extraversion can predict the level of gelotophobia while agreeableness can predict the level of katagelasticism, though emotional stability can predict the level of gelotophilia only for the typically developed group. This suggests that personality is not the reason for the level of gelotophilia in those with ASD.

Lastly, the mediation analysis revealed that the level of gelotophobia was completely mediated by extraversion, indicating that those in the ASD group had a higher level of gelotophobia than those in the typically developed group, which was mainly driven by extraversion. This finding supports the claim by Ruch and Proyer (2009b) that an individual with a lower level of extraversion is less adept at social interaction and is less able to engage in humor with others. This, in turn, makes it less likely for such individuals to experience the positive influence of laughter and humor. Moreover, they are more ill at ease in teasing situations and more afraid of being laughed at. In addition, extraversion was found to have an influence on the level of gelotophilia, although the mediated effect was partial. Those with higher levels of extraversion are keen to interact with others to create interesting situations, and even to engage in self-mockery. They do not feel embarrassed or uncomfortable at being laughed at; rather, they enjoy it (Ruch and Proyer, 2009a). Hence individuals with higher levels of extraversion usually have higher levels of gelotophilia. Further, the Big Five personality traits were found to confer no mediated effect on katagelasticism. We found that agreeableness inhibits the relationship between autism and katagelasticism, so that individuals with lower levels of agreeableness also have higher levels of katagelasticism (Ruch et al., 2013). As those with autism were found to have lower levels of agreeableness and also lower levels of katagelasticism than those in the typically developed group, this indicates that katagelasticism is not driven by personality but by other, unknown, factors.

Our results suggest that gelotophobia develops not from autism, but from a lower level of extraversion. For individuals with autism, a group therapy dealing with social skills (Glinski and Page, 2010) or a training program in emotional competencies will help (Nelis et al., 2011). Those with autism can learn interpersonal skills and thus learn how to engage in humor and how to cope with teasing situations. This, in turn, may alter their negative perception of interpersonal interaction, and thereby improve their fear of being laughed at.

Our findings suggest that future studies should investigate the reasons for katagelasticism and gelotophilia in order to understand what influences levels of gelotophilia and katagelasticism for those with ASD, and why those with ASD have both lower levels of agreeableness and lower levels of katagelasticism. They also suggest that it would be beneficial to create a comprehensive model of mockery styles based on the model of the putative causes and consequences of gelotophobia.

### Limitations

The present study has several limitations. First, we did not manipulate variables in the current study, so we cannot draw any inferences about the causal directions of the relationships. Titze (1995, 1996, 1997) considers that past experience of being laughed at during childhood influences personality development. More empirical studies of the relationships between gelotophobia and personality are needed, particularly longitudinal studies. Second, the present study used a test and a self-reporting questionnaire as research tools but lacked any other source of information; for example, observations by parents, teachers, or peers. Social desirability may have been a source of bias, indicating that more objective data should be used in future research. Third, the results of the current study were possibly restricted to males, because purposive sampling was used in this study. The gender ratio of individuals with autism in Taiwan is 8:1 (males to females), so the proportion of female participants in our study was only 13%. Therefore, if females and males had been included equally in the sample, it would have been necessary to examine gender as a factor. Fourth, the present study did not collect demographics of participants, e.g., parental socioeconomic status. Past research has indicated that a positive relationship has been observed between socioeconomic status and ASD prevalence (Durkin et al., 2010). Socioeconomic status might be a confounder of any associations between ASD status, personality traits, and gelotophobia-related traits. To investigate the index of socioeconomic status in future research, it would be more helpful to understand the causal mechanisms or confounding factors associated with ASD. Finally, because the participants of this study were Taiwanese students, the results may only be applicable to participants from an Asian cultural background. Thus, we recommend that future studies include participants from various cultures to examine whether the findings of this study differ culturally.

## Author contributions

L-PT and C-PA designed and conducted the research. M-NT and C-LW analyzed the data and drafted the manuscript under the supervision of H-CC. All authors approved the final version of the manuscript for submission.

### Conflict of interest statement

The authors declare that the research was conducted in the absence of any commercial or financial relationships that could be construed as a potential conflict of interest.
